# Revisiting the baby schema by a geometric morphometric analysis of infant facial characteristics across great apes

**DOI:** 10.1038/s41598-023-31731-4

**Published:** 2023-03-29

**Authors:** Yuri Kawaguchi, Koyo Nakamura, Tomoyuki Tajima, Bridget M. Waller

**Affiliations:** 1 Comparative Cognition, Messerli Research Institute, University of Veterinary Medicine Vienna, Vienna, Austria; 2 Japan Society for the Promotion of Science (JSPS), Tokyo, Japan; 3 Faculty of Psychology, Department of Cognition, Emotion, and Methods in Psychology, University of Vienna, Vienna, Austria; 4 Faculty of Science and Engineering, Waseda University, Tokyo, Japan; 5 Department of Anthropology, Washington State University, Vancouver, USA; 6 Unit of Synergetic Studies for Space, Kyoto University, Kyoto, Japan; 7 Evolution and Social Interaction Research Group, NTU Psychology, Nottingham Trent University, Nottingham, UK

**Keywords:** Developmental biology, Evolution, Psychology, Zoology

## Abstract

Infants across species are thought to exhibit specific facial features (termed the “baby schema”, such as a relatively bigger forehead and eyes, and protruding cheeks), with an adaptive function to induce caretaking behaviour from adults. There is abundant empirical evidence for this in humans, but, surprisingly, the existence of a baby schema in non-human animals has not been scientifically demonstrated. We investigated which facial characteristics are shared across infants in five species of great apes: humans, chimpanzees, bonobos, mountain gorillas, and Bornean orangutans. We analysed eight adult and infant faces for each species (80 images in total) using geometric morphometric analysis and machine learning. We found two principal components characterizing infant faces consistently observed across species. These included (1) relatively bigger eyes located lower in the face, (2) a rounder and vertically shorter face shape, and (3) an inverted triangular face shape. While these features are shared, human infant faces are unique in that the second characteristic (round face shape) is more pronounced, whereas the third (inverted triangular face shape) is less pronounced than other species. We also found some infantile features only found in some species. We discuss future directions to investigate the baby schema using an evolutionary approach.

## Introduction

All mammals, and humans in particular, have a long period of vulnerability in early development during which extensive care from adults is critical for survival. The mechanisms underlying how adults interact with infants are therefore important to understand. In humans, infant faces convey rich information including health, age, and sex^1^, and are highly effective at capturing visual attention^2,3^. They are also generally perceived as highly attractive, induce positive emotions and parenting motivation^4–7^, and activate the medial orbitofrontal cortex of the perceiver, which is implicated in reward behaviour^4,5^. Therefore, they play a key role in human parental investment^1,6^. Infant faces, however, are not simply miniature adult faces. Almost 80 years ago, the prominent ethologist Konrad Lorenz proposed the “baby schema^8^”, a set of physical features of infant faces, including a prominent forehead, bigger eyes located lower in the face, protruding cheeks, and bodily features such as a relatively large head. The famous illustrations of the baby schema by Lorenz depict not only humans, but also hares, dogs, and birds. Since then, this baby schema has been assumed to be shared across species and induce caretaking behaviour from conspecific adults^8,9^. A number of empirical studies in humans have supported this idea. For example, exaggeration of the baby schema increases the attractiveness of faces and parental motivation^10–13^. Neurological studies have also found that in nulliparous women the baby schema activates the nucleus accumbens, which mediates reward processing^14^. This evidence suggests that, at least in humans, the baby schema is a salient positive stimulus with a robust perceptual impact.

There is some evidence that an infantile appearance in non-human animals may be similarly related to caretaking behaviour^9,15–18^, but this has been tested exclusively in human perceivers. There are also some studies manipulating the extent of babyness in animal faces and testing the effect of the manipulation on cuteness evaluation by humans^16,19^, but the manipulations were based on developmental changes in human faces and were not species-specific. Thus, the faces were made to look more babylike from a human perspective, which may or may not reflect facial immaturity in each species.

The first step is to identify whether a common baby schema exists across species in terms of appearance. Craniofacial development has been well-studied among primate species including humans. For example, chimpanzees develop elongated braincases while humans develop globular ones and they do much faster and for longer. Chimpanzee faces grow more projecting while humans faces grow mainly vertically^20^. Nevertheless, if one is interested in faces, which are seen by others and potentially function as social cues or signals (as the baby schema is supposed to), studying faces with muscle and soft tissue is necessary. As far as we know, there is no systematic study quantifying and comparing shared infant face characteristics among species. This is surprising as it has been claimed that the baby schema “seems to be a universal stimulus^21^”. It is possible that the existence and function of the baby schema (i.e. inducing parenting) evolved only in humans as human infants are costly to raise and are born at a very early stage of development. Alternatively, the baby schema could be shared and function similarly across evolutionarily related species. Therefore, it is important to study first if baby schema exists in our closest relatives to understand the evolution of the mechanisms underlying human parenting.

The present study investigated which facial characteristics are shared across five species of great apes (including humans) using geometric morphometric analysis. Our first and main goal was to examine and update the classic description of the baby schema. We targeted great apes to compare the similar facial morphology of these phylogenetically close species. A second goal of the study was to examine any variation in the characteristics of infant faces across different species. A previous study revealed that people perceive infants of non-mammals requiring parental care (i.e. semiprecocial species) as cuter than those exhibiting no parental care (i.e. superprecocial species)^9^. Thus, it is possible that species may also show different kinds or/and degrees of infantile face features according to socio-ecological factors such as the extent of alloparenting. We, therefore, aimed to develop a method to capture any variation in infantile facial characteristics across great ape species for future analysis by using geometric morphometrics. Geometric morphometrics is a data-driven approach. It does not require any predetermined assumptions (about adult/infant differences in our case) and instead measures the relative shape differences between the two categories. In previous face studies, geometric morphometrics have been used to quantify skeletal craniofacial morphology e.g.^22^, but it has also been used for living faces with full soft tissue (humans e.g.^23^ and non-human animals e.g.^24^).

## Methods

### Data analysis

We analysed frontal facial images of five species of great ape: humans (*Homo sapiens*), chimpanzees (*Pan troglodytes*), bonobos (*Pan paniscus*), mountain gorillas (*Gorilla beringei beringei*), and Bornean orangutans (*Pongo pygmaeus*). Although gorillas and orangutans consist of two and three species respectively and express morphological differences^25,26^, we analysed only one species for each due to availability of the pictures. Chimpanzees were western chimpanzees (*Pan troglodytes verus*) with one infant who was a hybrid between a western chimpanzee and a central chimpanzee (*Pan troglodytes troglodytes*). Those images were taken either in the wild or in captivity by authors, other researchers, or photographers (see Table A.1 for more details). The images of humans were from open-access databases^27,28^, and the reported ethnicity of those is all white. Sixteen frontal images for each species (eight for adults and infants respectively), totalling 80 images were analysed. Although larger sample size would be ideal, eight images were chosen as a maximum to ensure parity across species as taking fully frontal photos of infant faces is extremely difficult due to lack of independence from the mother. In a previous study^29^ which analysed shape differences between adult and infant chimpanzee faces in the similar way, the number of images was the same. Four out of eight images were male for each species and age category with one infant gorilla whose sex was unknown. The average age of adults was 19.6 years old (bonobos (mean ± SD): 19.9 ± 4.7, chimpanzees: 18.4 ± 2.7, humans: 20.4 ± 1.8, gorillas: 18.6 ± 5.2, orangutans: 20.8 ± 3.15) while that of infants was 6.6-months-old (bonobos: 6.9 ± 2.5, chimpanzees: 6.8 ± 2.6, humans: 6.6 ± 2.3, gorillas: 6.6 ± 2.3, orangutans: 6.1 ± 2.7). Although there are slight differences in developmental speed among species (e.g. age of weaning or sexual maturation), infants of this age are at least not neonate anymore but much younger than weaning age, and adults of the targeted age are all sexually mature in each species. The criteria to choose the facial images were (1) does not exhibit clear facial movement, (2) mouth is closed, (3) both eyes are open, and (4) chin line is visible.

**Figure 1:**
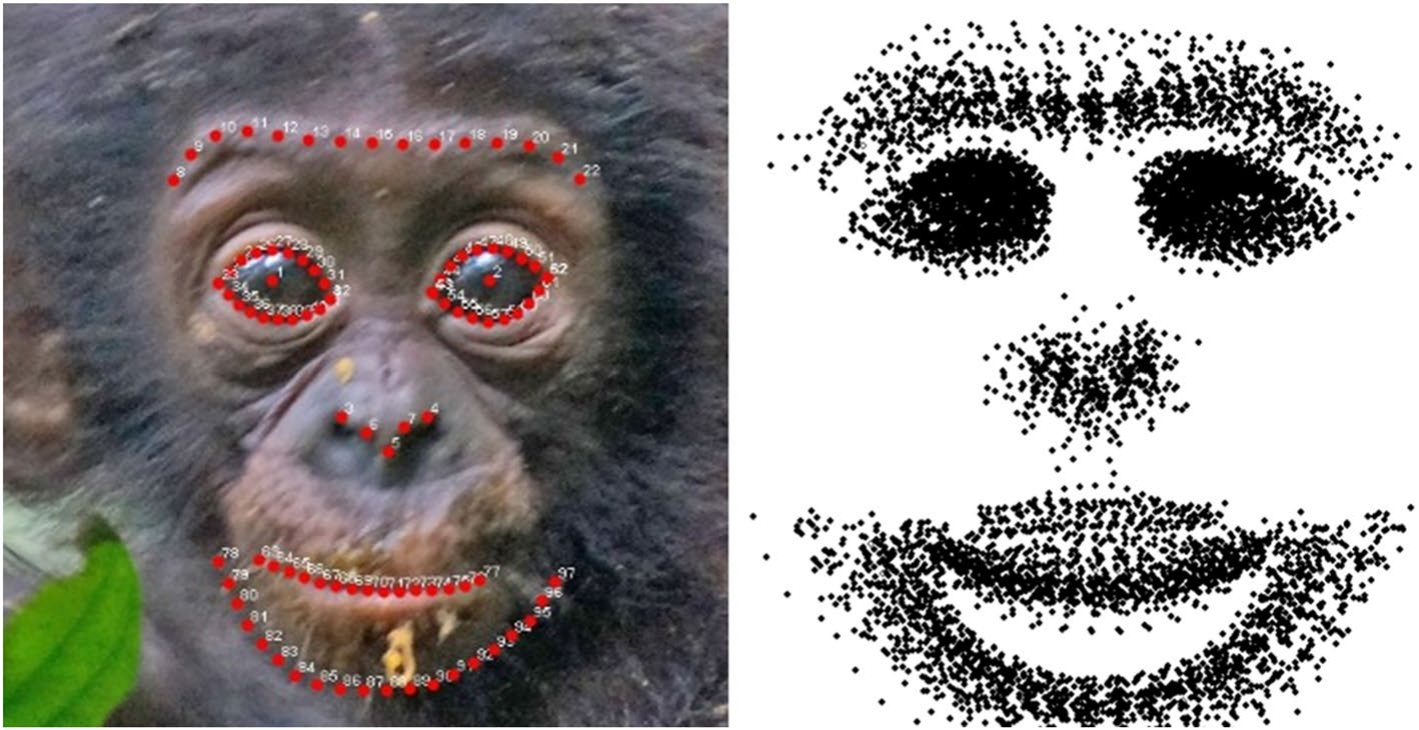
An example of landmark delineation on an infant bonobo face (left) and the superposition of all landmarks of 80 faces after the Procrustes fit for visualization of the whole variation (right).

Ninety seven landmarks were manually placed on each face by one of the authors (Y. K.) with tpsDig2 software (version 2.31)^30^. Since we did not aim to analyse colour (and all human infant images were black and white), we changed the brightness and contrast of the images to achieve maximum visibility. Landmarks delineated the supraorbital torus, eye outlines, pupils, nose edges, mouth, and chin based on human face morphological studies^23,31^ with some modification to apply non-human primate faces (i.e. landmarks on supraorbital torus and oral commissure instead of eyebrows and lip) (Fig. 1). Out of 97 landmarks, 76 were designated as semi-landmarks. Semi-landmarks denote curves and outlines while other landmarks are represented as points that are geometrically homologous (e.g. mouth corner) among specimens. We did not include face outlines besides the chin because it is difficult to consistently identify the face outline due to facial hair in non-human primates. The landmarks and the slider file used for the definition of semilandmarks are available in Supplemental Materials. In order to confirm the reliability of the landmark annotation, the same person annotated 10 images (12.5%) randomly chosen from the dataset and intra-annotator reliability for all 97 landmarks (x–y coordinates) was calculated. The Inter Class Correlation Coefficient was excellent (x-coordinates: mean 0.994 (0.975–0.998), y-coordinates: mean 0.997 (0.986–0.999)).

Average adult and infant faces across all species and specific to each species were generated by tpsSuper (version 2.06)^30^ for the purpose of visualization of shared morphological traits. All the landmarks of the images were superimposed by a generalized Procrustes analysis and analysed by a relative warp analysis with tpsRelw software (version 1.75)^32^. By using Procrustes analysis all the images were aligned in regard to orientation and size to get maximum fit. Then, a principal component analysis (PCA) (i.e. a relative warp analysis) was performed, which detects the main components contributing to the variation of landmark configuration among images. A relative warp analysis yielded 79 principal components and among them, the first 11 PCs accounted for more than 95% morphological variation of all 80 images (Table A.2).

In order to examine if these characteristics are reliable features to differentiate two age categories from faces, we tested if the age category (infant vs. adult) is correctly classified based on PC scores by using classification algorithms. Our approach is analogous to a previous study that used the classification performance of machine learning in order to evaluate if there is potential information in face traits to determine certain attributes (e.g. sex or age) among the twelve species of guenons (*Cercopithecini*)^33^. There are several methods which are used to make classifications in morphology studies^34^. Thus, we fitted four different classification models with scores of all PC1-11, and chose the best one based on a classification performance^35^. The four models are: (1) linear discriminant analysis (LDA, or discriminant function analysis), (2) linear support vector machine (SVM) without hyperparameter tuning, (3) linear SVM with hyperparameter tuning, and (4) non-linear SVM (with Radial Basis Function kernel) with hyperparameter tuning. LDA and SVM are both algorithms which build a decision boundary between two classes (*adults and infants* in our case) in dimensional space of data (*scores of target PCs* in our case). The decision boundary is determined by distribution of the data in LDA while it is determined by points that are close to the other class in SVM. For SVM models with hyperparameter tuning, hyperparameters (the optimal cost and gamma) were determined by a grid search from 2^–10^ to 2^10^ with a fourfold cross validation. As an index of classifier performance, we used the *accuracy* and *area under curve* (AUC). The accuracy is the percentage of correct prediction, and the area under receiver operating characteristic curve (hereafter just AUC) is a parameter which takes into account both false-positive rate and true positive rate. AUC can take the value from 0.5 (i.e. a random classifier) to 1 (i.e. a perfect classifier). All the modelling was conducted with open-source Python (version 3.8.10) Scikit-Learn packages.

Then we remodelled age category classification with the best model based on each of PC 1–11 for all the five species pooled together and for each species separately to see which PCs are key features to differentiate adult and infant faces. In order to compare the model performance with random classification, we conducted 1000 permutation tests. Based on the p-value of the permutation tests, we decided if each PC is reliable cue to differentiate age categories.

## Results

**Figure 2:**
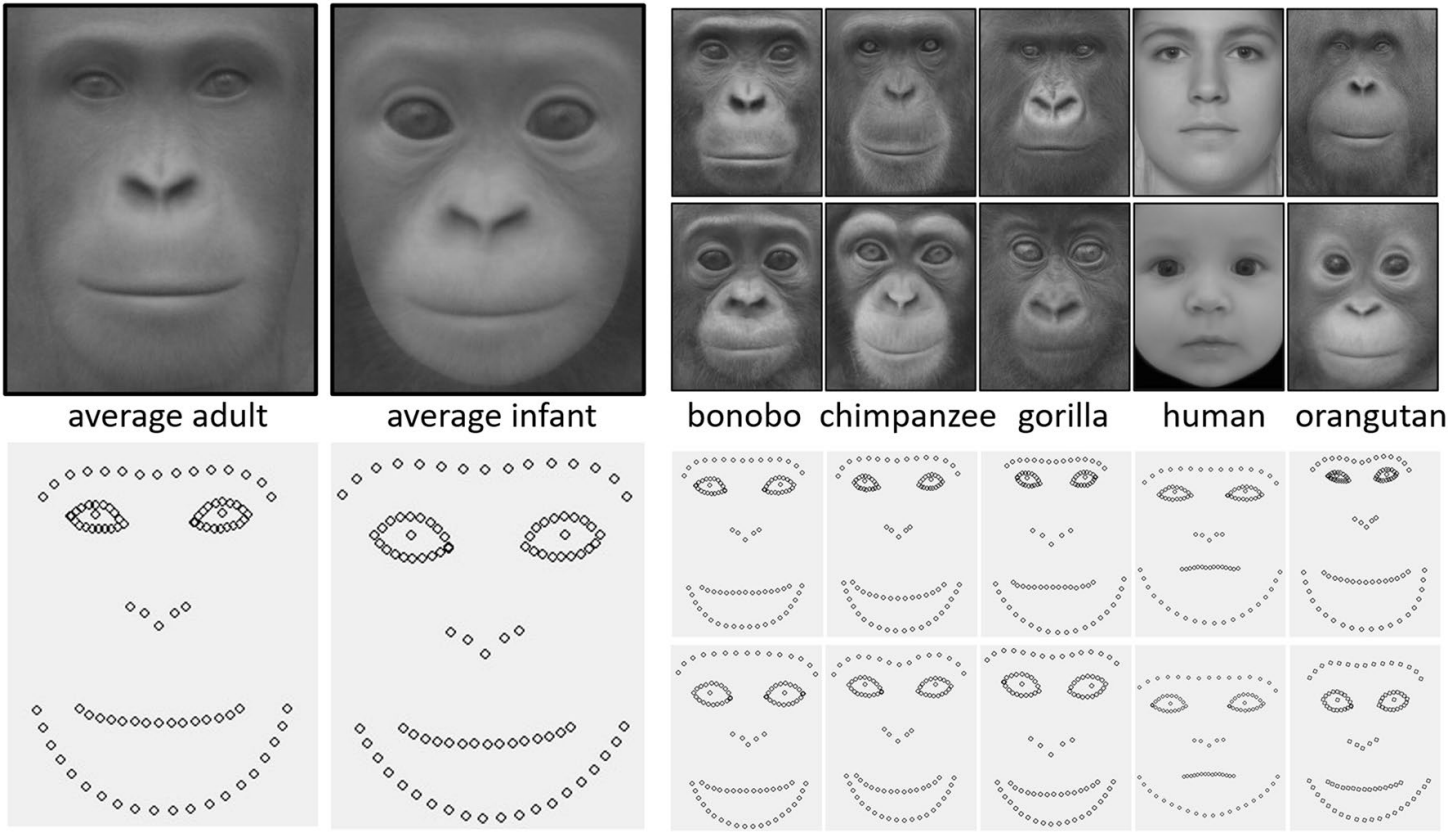
Average adult and infant faces and landmark configurations among five species, and those of each species (top: adults, bottom: infants).

Figure 2 illustrates average adult and infant faces and landmark configurations across the five species, and those of each species. Among four models, the tuned non-linear SVM was selected based on the highest accuracy and AUC of classifying age categories when all species pooled (LDA: acc. 0.8, AUC 0.91, untuned linear SVM: acc. 0.78, AUC 0.95, tuned linear SVM: acc. 0.94, AUC 0.98, tuned non-linear SVM: acc. 0.95, AUC 0.98). Thus, we performed the further analysis with the tuned non-linear SVM. We then checked classification performance and conducted a permutation test when each PC was used alone. Age classification was performed with high reliability on the basis of PC 1 (acc. 0.81, AUC 0.90, *p*-value of permutation test < 0.01), PC 2 (acc. 0.78, AUC 0.87, *p* < 0.01), PC 3 (acc. 0.61, AUC 0.63, *p* = 0.01), or PC 11 (acc. 0.61, AUC 0.63, *p* = 0.04) when all species pooled (i.e. 80 faces).

**Figure 3:**
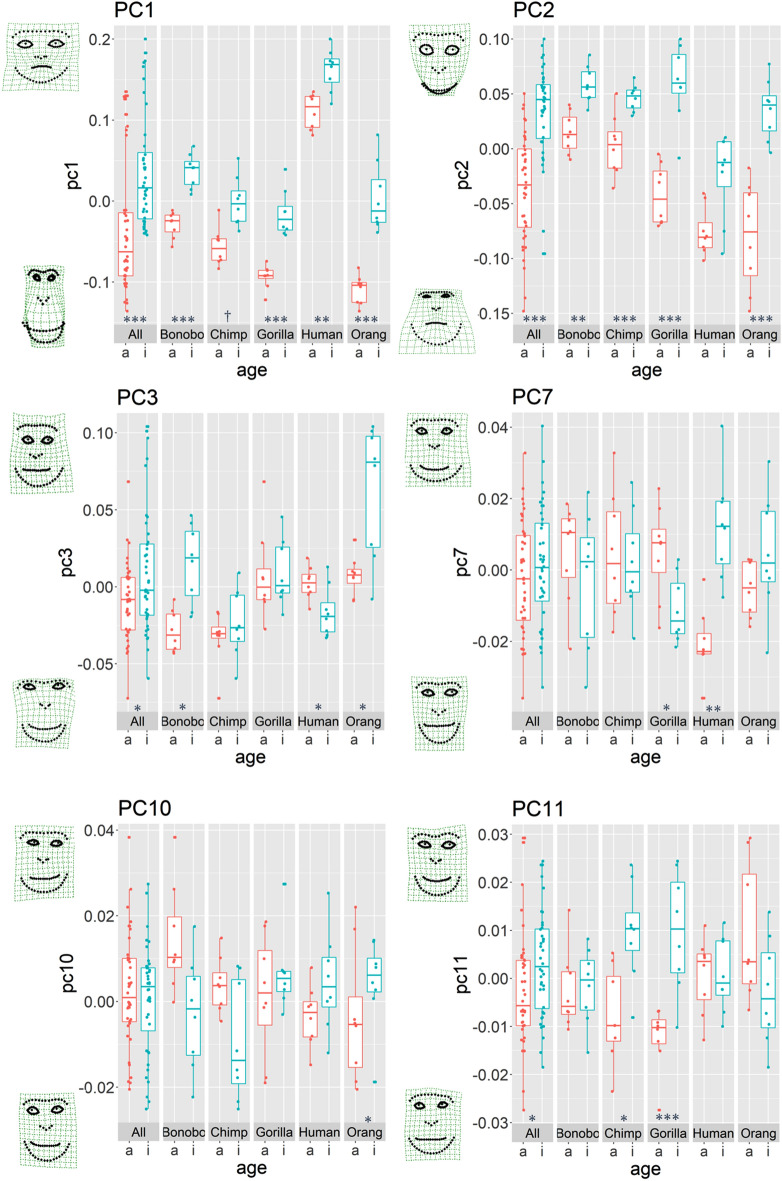
Scores of PCs. Asterisk indicates significant difference of the score between adult and infant faces by permutation tests (***p < 0.005, **p < 0.01, *p < 0.05, † < 0.10). Pink plots indicate adult images while blue plots indicate infant images. Faces represent landmark configurations of high (+ 1.5 SD) and low (− 1.5 SD) PC scores.

Next, we conducted age classification for each species separately based on each of PC 1- 11 in order to check if there are any species differences. First, we found that PC 1 is a reliable age class predictor and scores are higher in infants than in adults across all species except chimpanzees (*p* = 0.07, others *p* < 0.01. Figure 3 and Table 1, see also Fig. A.1 and Table A.3 for details). By visually inspecting the exaggerated facial shape that each PC represents, PC 1 seemingly represents facial roundness and relative eye size; a high score indicates the face has a round and short shape on the vertical axis with relatively bigger eyes. The PC 2 is also a reliable predictor and the scores are higher in infants than in adults across all species except humans (*p* = 0.11, others *p* < 0.01). PC 2 represents the holistic configuration of the face, and a high score indicates a top-heavy (i.e. inverted triangle) face shape with relatively bigger eyes. PC 3 is a reliable predictor only in bonobos, orangutans, and humans (all *p* < 0.05). However, the direction is not consistent. The scores of PC 3 are higher for infants in bonobos and orangutans, but higher for adults in humans. PC 3 seemingly represents the distance between eyes and between eyes and nose, a high score indicates a centripetal face (i.e. the distance between those features is close). We also found that PC 7 is a reliable predictor in gorillas and humans (*p* < 0.05). The direction is again different from each other. In gorillas, adults score higher, while in humans, infants score higher. Based on visual inspection, PC 7 likely represents chin shape; high scores indicate a horizontally wider and vertically shorter chin. Moreover, PC 10 is a reliable predictor only in orangutans; infants score higher than adults (*p* < 0.05). However, this component seemingly represents just an artifact, namely lateral asymmetry. Lastly, PC 11 is a reliable predictor only in two species; the scores are significantly higher for infants than adults in chimpanzees and gorillas (*p* < 0.05). PC 11 likely represents the shape of the supraorbital torus; a high score indicates a curved instead of straight one. The other PC 4, 5, 6, 8, and 9 did not contribute to age classification in any species. All the visualization and the scores of PC 1–11 were shown in Fig. A.2 and Figs. A.3-A.8.

**Table 1:** Significant PCs and the directions (***p < 0.005, **p < 0.01, *p < 0.05, † < 0.10).

	PC 1	PC 2	PC 3	PC 7	PC 10	PC 11
All species	A < I***	A < I***	A < I*			A < I*
Bonobo	A < I***	A < I**	A < I*			
Chimpanzee	A < I†	A < I***				A < I*
Gorilla	A < I***	A < I***		I < A*		A < I***
Human	A < I**		I < A*	A < I**		
Orangutan	A < I***	A < I***	A < I*		I < A*	

PC 1 and PC 2 can be described as shared infant face features because they are reliable predictors of age category in most of the species, and the tendency between adults and infants is consistent among all species. That is, infants in general have a vertically short (PC 1) and top-heavy (PC 2) face shape with relatively bigger eyes (PC 1 and 2). Nevertheless, there are also species differences in the robustness of shared infant features described by PC 1 and PC 2, although we did not compare PC scores themselves across species with statistical analysis. For example, scores of PC 1 in humans (both adults and infants) stood out among five species (Fig. 3), indicating that human faces are generally rounder compared with other species. For PC 2, human infants have lower scores compared with infants of other species (Fig. 3), which means human infant faces have a more bottom-heavy (i.e. triangular) configuration compared with infants of other species.

## Discussion

As far as we know, this is the first attempt to examine and update the classic work on the baby schema by Lorenz^8^ using a data-driven approach. The facial shape analysis showed that there are shared infantile face features across species. Based on our results, infant faces are defined by three face characteristics in all five species; (1) relatively bigger eyes located lower in the face (PC 1 and 2), (2) a rounder and shorter (on the vertical axis) face shape (PC 1), and (3) an inverted triangular face shape (PC 2). These characteristics are consistent and robust among the five species. When we compare these characteristics and those originally mentioned as baby schema by Lorenz^8^, the first characteristic about eyes was clearly mentioned as one of the features of baby schema. Not particularly rounder face shape (PC 1), but rounder body shapes, in general, was also listed as a characteristic of baby schema. The second characteristic, an inverted triangular face shape itself was not listed by Lorenz, but it may, at least partly, be corresponding to a protruding forehead, one of the characteristics of baby schema. Besides these features, the assumption that infants have relatively smaller noses and mouths was not clearly listed as the original baby schema by Lorenz^8^, but often mentioned as the baby schema in later literature e.g.^19^. However, we did not find clear evidence supporting this at least from the present study.

These infantile face features seen in all species might reflect physical constraints such as differential timing of development of each face part. For example, the development of eye growth ceases much earlier than that of other parts of the face, resulting in relatively larger eye sizes in young faces^20^. The protruding forehead is seen in infants because it accommodates the relatively large size of the brain, and faces experience vertical growth later^20^. The infants’ short and top-heavy faces in our findings could be probably explained by these processes. Although PC 1 and PC 2 are both related to bigger eyes, each PC is orthogonal to the other, meaning PC 1 and PC 2 are reflecting independent characteristics. Bigger eyes are not expressed alone but accompanied with other features (rounder or top-heavy face shape). Our findings may explain the reason why bigger eyes of infants alone poorly contribute to cuteness perception in humans^36^.

Although these face features are shared by infants among species, there seem to be species differences in the extent to which these differences manifest. Human infant faces are especially unique compared to similar-aged infants of other species, which is consistent with the findings from a previous cranial study^20,37^. First, human infants look immature with respect to one component (PC 1), facial roundness. This may be related to Lorenz’s argument that compared with human infants, the baby schema is *less* embodied in young non-human primates, who have “long legs, long snout, and sunken cheeks and they appear cute to very few people”^8^. Roundness in human faces even in adults may reflect neoteny, where humans retain immature features including feminized or juvenilized morphology^38^. Conversely, human infant faces, compared with other species infant faces, score lower in PC 2, which means that they tend to be bottom-heavy rather than top-heavy. One can say that in this regard human infant faces look more mature than other species. Nevertheless, it is also possible that bottom-heavy characteristic of human infants may reflect chubby cheeks, which is probably uniquely present in humans as Lorenz pointed out^8^ due to greater adipose tissue in the face^39^.

While it is obvious that PC 1 and 2 are related to developmental face change in great apes in general, three other PCs indicate species-specific infant facial features because the relationship between infants and adults varied across species. First, regarding PC 3, infant faces score higher than adult faces in bonobos and orangutans (i.e. infant faces are more *centripetal* than adult ones). On the other hand, the scores of PC3 are lower in human infants (i.e. infant faces are more *centrifugal* than adult ones). The results are consistent with a previous human study which found that human faces with wide eyes are perceived as young^40^. However, this is specific to humans (at least among great apes). Second, human infants have a horizontally wider and vertically shorter chins than adults while gorilla infants have narrower and longer chins (PC 7). Human infants have a wide posterior dental arcade compared with infants of *Pan* species^41^. Moreover, the chin develops prominently in humans^20^ and can be considered a uniquely human characteristic. These factors may be related to the results of PC 7. Lastly, only chimpanzee and gorilla infants have curved supraorbital torus (PC 11). The characteristics defined by those PCs are infantile characteristics only in certain species. Thus, we should be cautious to assume that infant faces have the same characteristics across species.

An important next question is what is the function of infantile face features in non-human primates, if any? In humans, a large body of literature supports the hypothesis that infantile face features induce cuteness perception and parenting motivation^4,5,7,11,42^. Such features are, therefore, likely to contribute to infant survival. These features may function similarly in non-human primates^8,9^. Moreover, paedomorphic appearance in infants and caregiving behaviour may have coevolved in primates or other orders^9^. It is beyond the scope of the current study to address this question, but future studies should address this point. One way to test this may be to use an index which evaluates the face immatureness of infants of the species and examine the relationship between face immatureness and other socio-ecological factors. Although from the present study it is not fully clear how much differences exist in the extent of face immatureness within infants of the non-human primate species, further investigation may reveal more. One of the predictions is that if the face immatureness encourages conspecifics to take care of infants as has been suggested, degree of (allo)parental care will be positively correlated with the face immatureness of the species. A related prediction is that if the baby schema encourages adults’ protective behaviour toward infants at risk as suggested for another infantile features^43^, infanticidal risk and face immatureness will also be positively correlated.

It should be noted that specific face morphology is not the only visual characteristic of infants. For example, some primate species have conspicuous coloration during infancy^43–46^. In orangutans, for example, skin colour around the eyes and mouth is bright during infancy while adults have darker skin^47^. Similarly, in chimpanzees, infants have paler face skin colour compared with adults and this infantile face colour is perceived by chimpanzees as a more salient cue than infant face morphology^29^. The potential functions of such infantile coloration in general (e.g. encouraging alloparenting^44,45^) have been suggested but are still under debate. At least for chimpanzees and orangutans among the species we analysed, the other facial cues showing “babyness” seemingly exist, so how much facial morphology alone plays a role is unclear. If species with infantile coloration have more (or less) morphological immatureness in faces to see whether these features are functionally related.

It is also informative to ascertain if and how individual differences in infant faces are related to other factors (e.g. health or the amount of care they receive from other individuals) although a larger sample size is necessary to test this. It is possible that infant facial cues are “relatively honest signs of fitness and health of infants^38^”. Indeed, humans, especially females, are very sensitive to subtle differences in infant faces^48,49^, and perceived cuteness of infant faces is correlated with perceived health^50,51^ and the quality of maternal care toward the infant^52^. Individual differences in infant faces can be important information for non-human parents as well because they need to decide how much investment they should allocate to their offspring^53^.

There are several limitations in this study. First, we analysed only specific morphological features of 2D faces. There is also a trade-off between including various landmarks in morphologically different species in analysis and setting corresponding landmarks consistently among them. Thus, it should be noted that our analysis does not cover all the features of facial morphology. Second, we did not control the living environment (wild versus captive) of the individuals we analysed. It is possible that the environmental factors, including food availability, affect face morphology during development. Third, the number of samples we analysed was small due to the difficulty of getting full-frontal face photos of non-human primate infants. Thus, we cannot fully rule out random variations in face photographs by artifacts such as lateral asymmetry captured by PC10. Nevertheless, at least the shared infant face features we reported here, namely PC 1 and 2, are robust and seem not to be artefactual. Lastly, this study focused on only five species of great apes although the concept of a baby schema has been applied to various species beyond primates. Our method using a geometric morphometric should be applicable to other species, especially primates, so future studies should include more species in order to ascertain how broadly infantile face features are shared. Regardless of those limitations, our study provides new insight regarding the evolution of paedomorphic appearance in infants. This is the first quantitative evidence that there is a “baby schema” which is shared across our closest relative species. In conclusion, some face features are indeed shared among great ape species but there are also significant species differences. The current study should be a good starting point to reveal how infantile visual features have played a role in social interaction over the course of mammalian evolution.

## Supplementary Information

Supplementary Figures.

Supplementary Information.

## Data Availability

Some of the analysed images owned by one of the authors, all the landmark information, and the slider file are available on Mendeley Data (http://dx.doi.org/10.17632/8hs593cyc2.1). The data associated with this research are available as supplementary materials.
