# Supplementary material for: Revisiting the baby schema by a geometric morphometric analysis of infant facial characteristics across great apes
Source: Sci Rep. 2023 Mar 29;13:5129. doi: 10.1038/s41598-023-31731-4 (PMC10060388; doi:10.1038/s41598-023-31731-4)
Supplement: Supplementary file 1 — Supplementary Figures. [file 41598_2023_31731_MOESM1_ESM.docx]

**Supplementary material**

*Revisiting the baby schema: a geometric morphometric approach reveals shared infant facial characteristics across great apes*

Yuri Kawaguchi, Koyo Nakamura, Tomoyuki Tajima, Bridget Waller


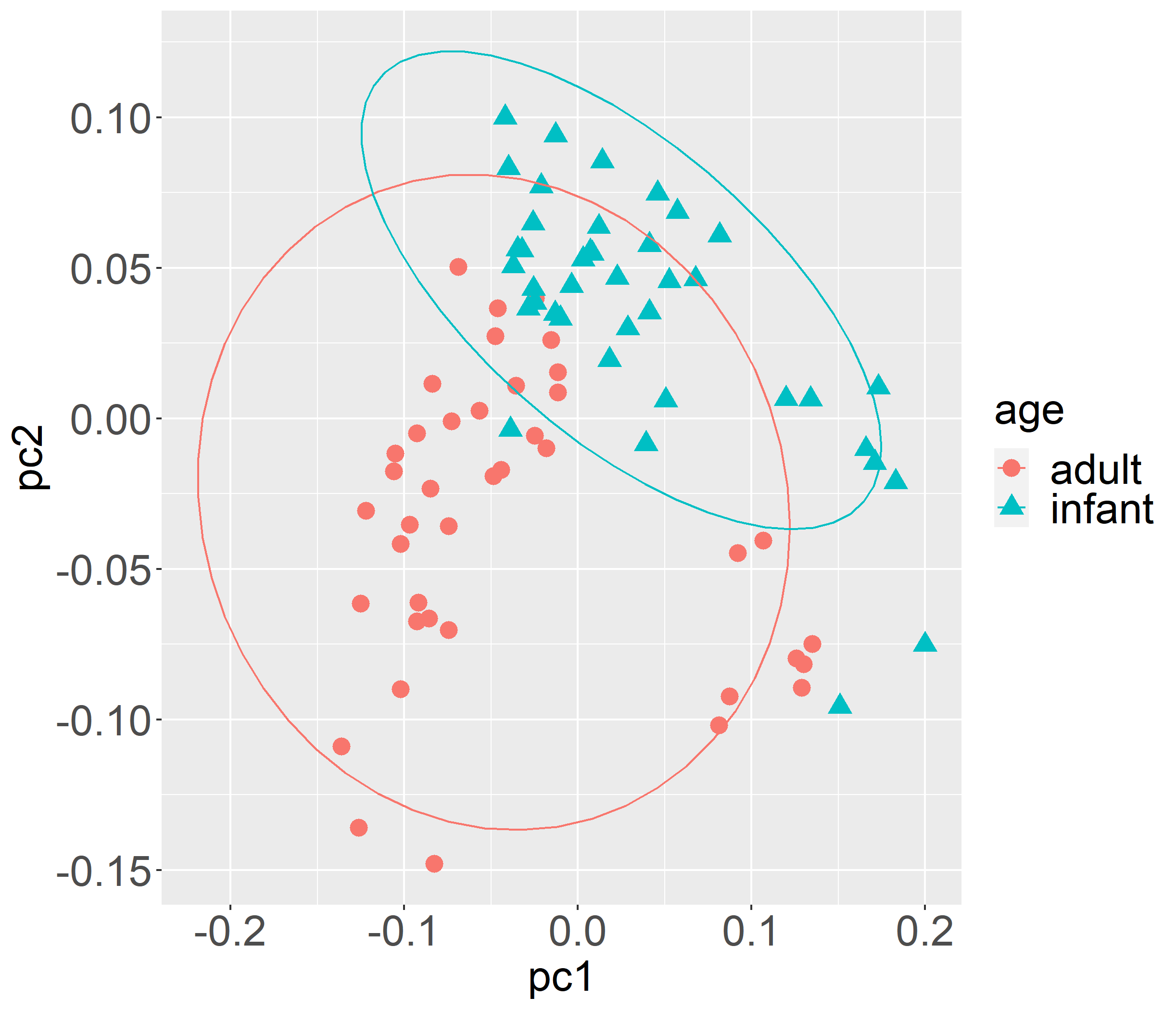

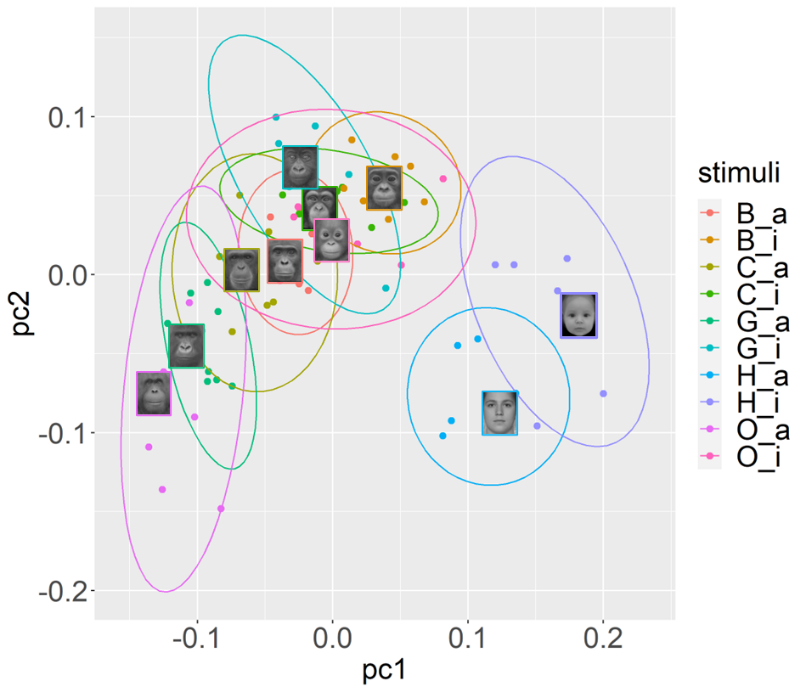


Fig. A.1. Scores of PC 1 and 2 for each face.


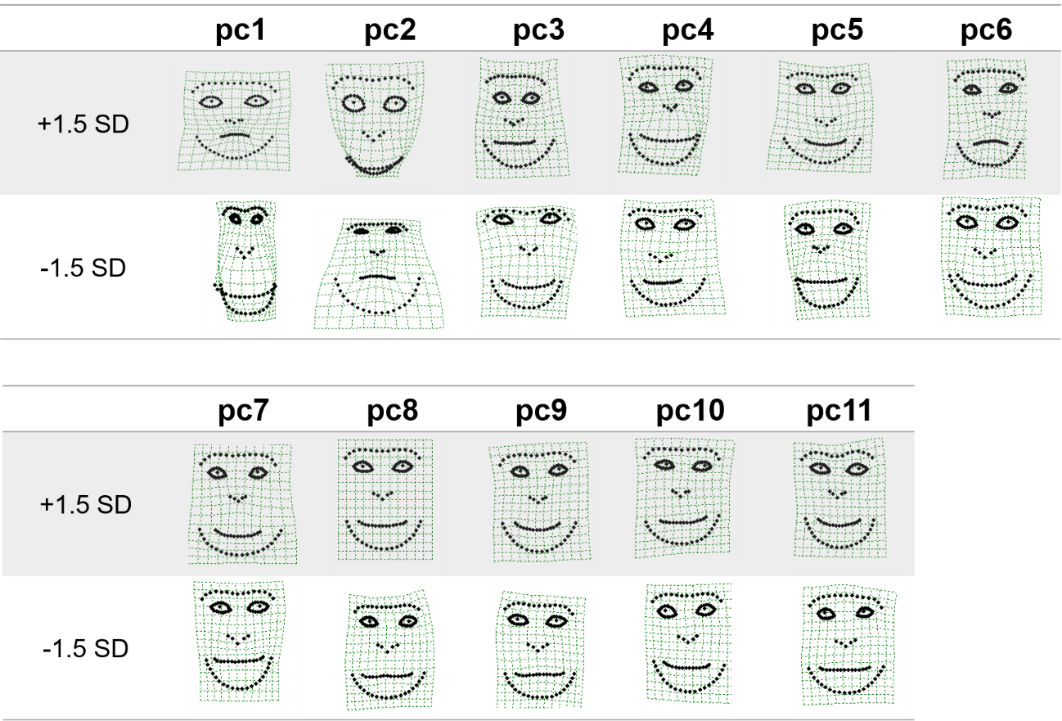


Fig. A.2. The landmark configurations of high (+1.5 SD) and low (-1.5 SD) PC scores (PC1-11).


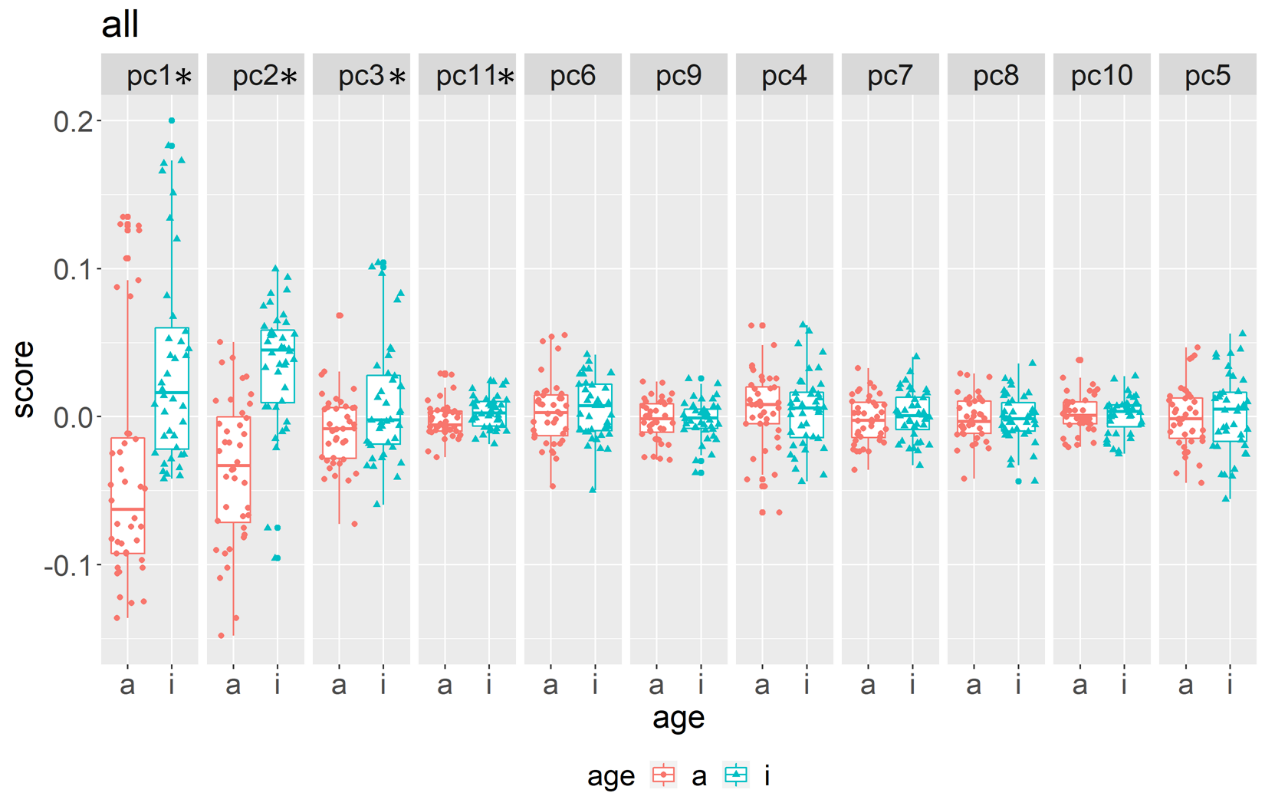


Fig. A.3. PC scores of all species.


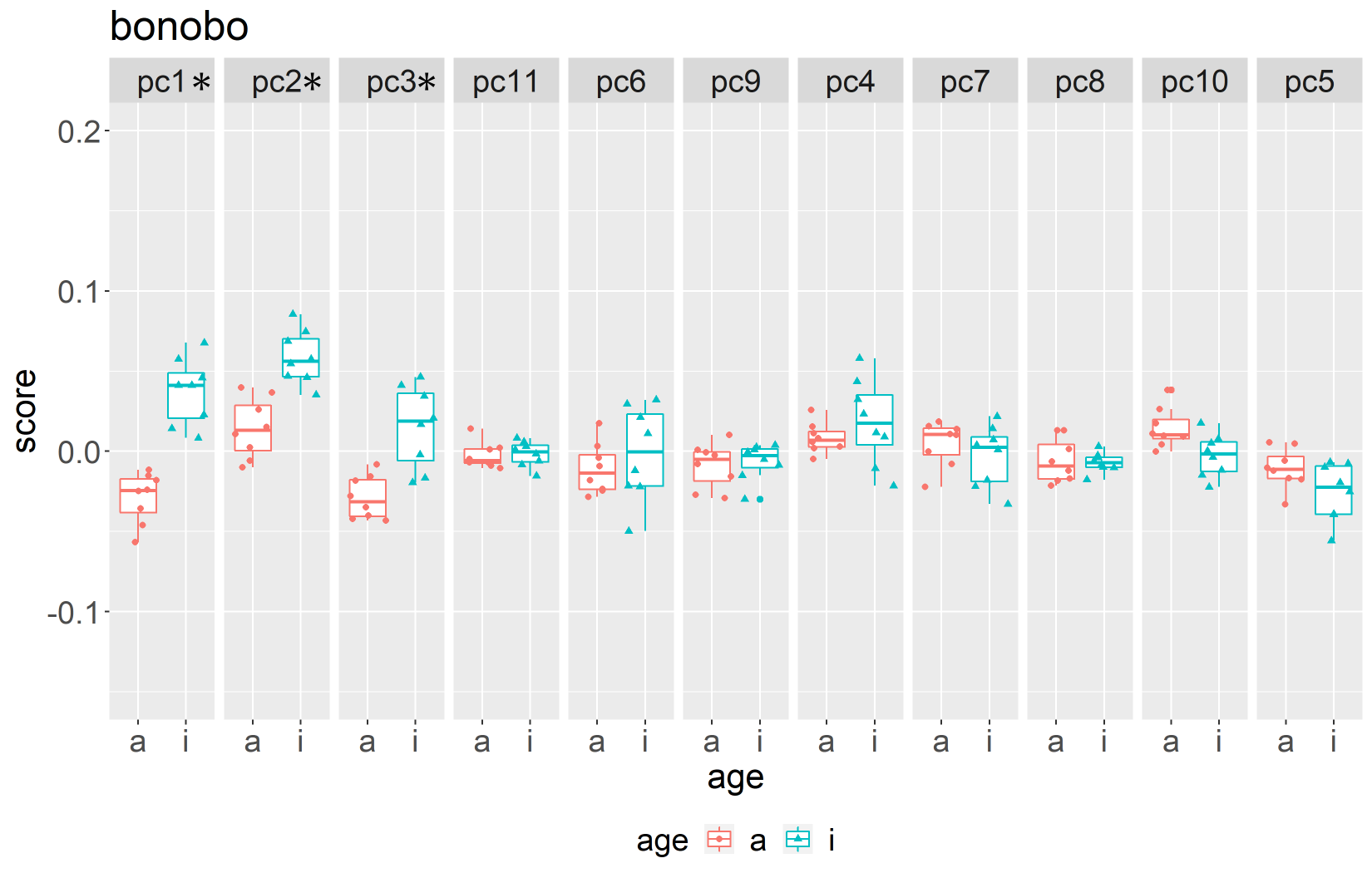


Fig. A.4. PC scores of bonobos.


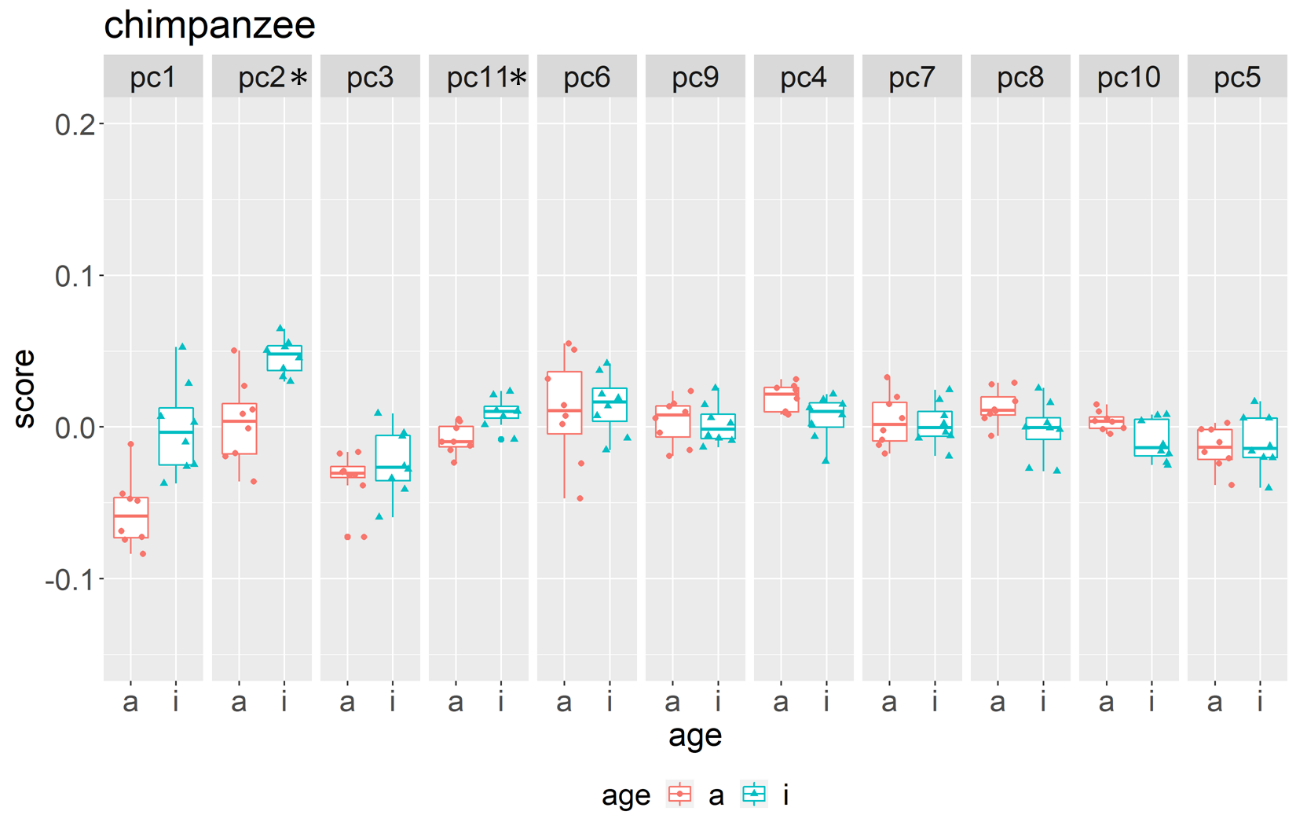
Fig. A.5. PC scores of chimpanzees.


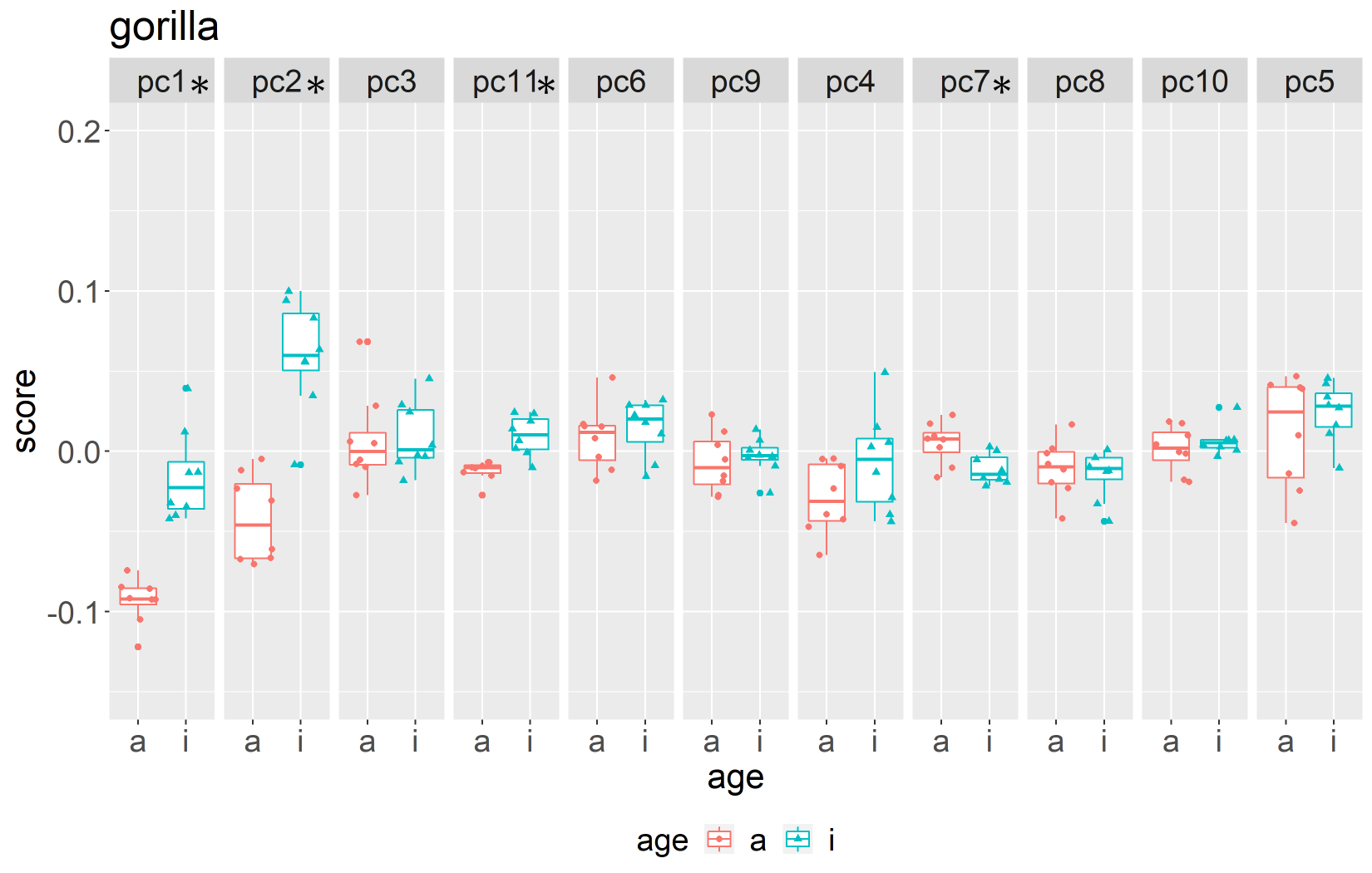


Fig. A.6. PC scores of gorillas.


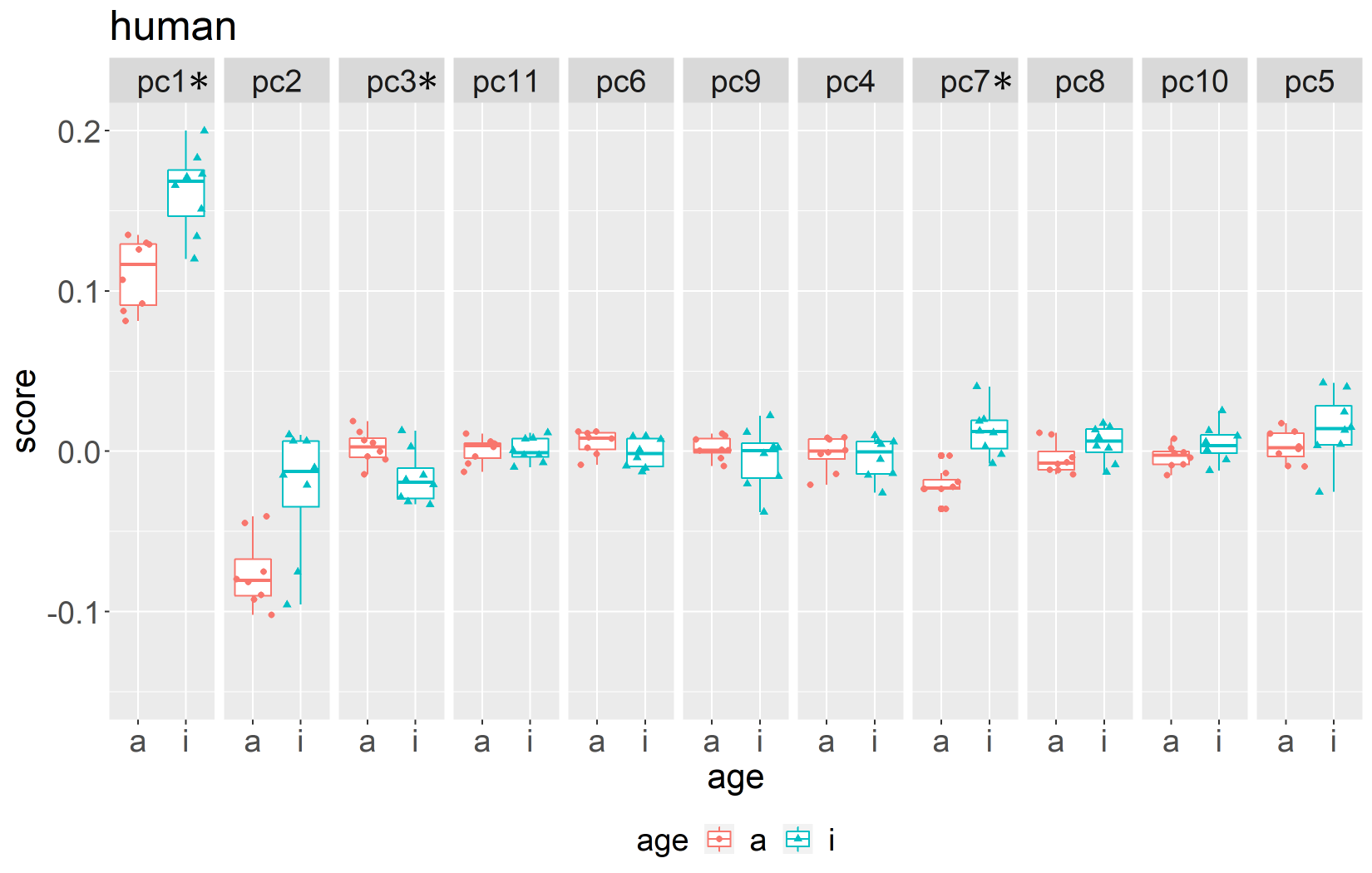


Fig. A.7. PC scores of humans.


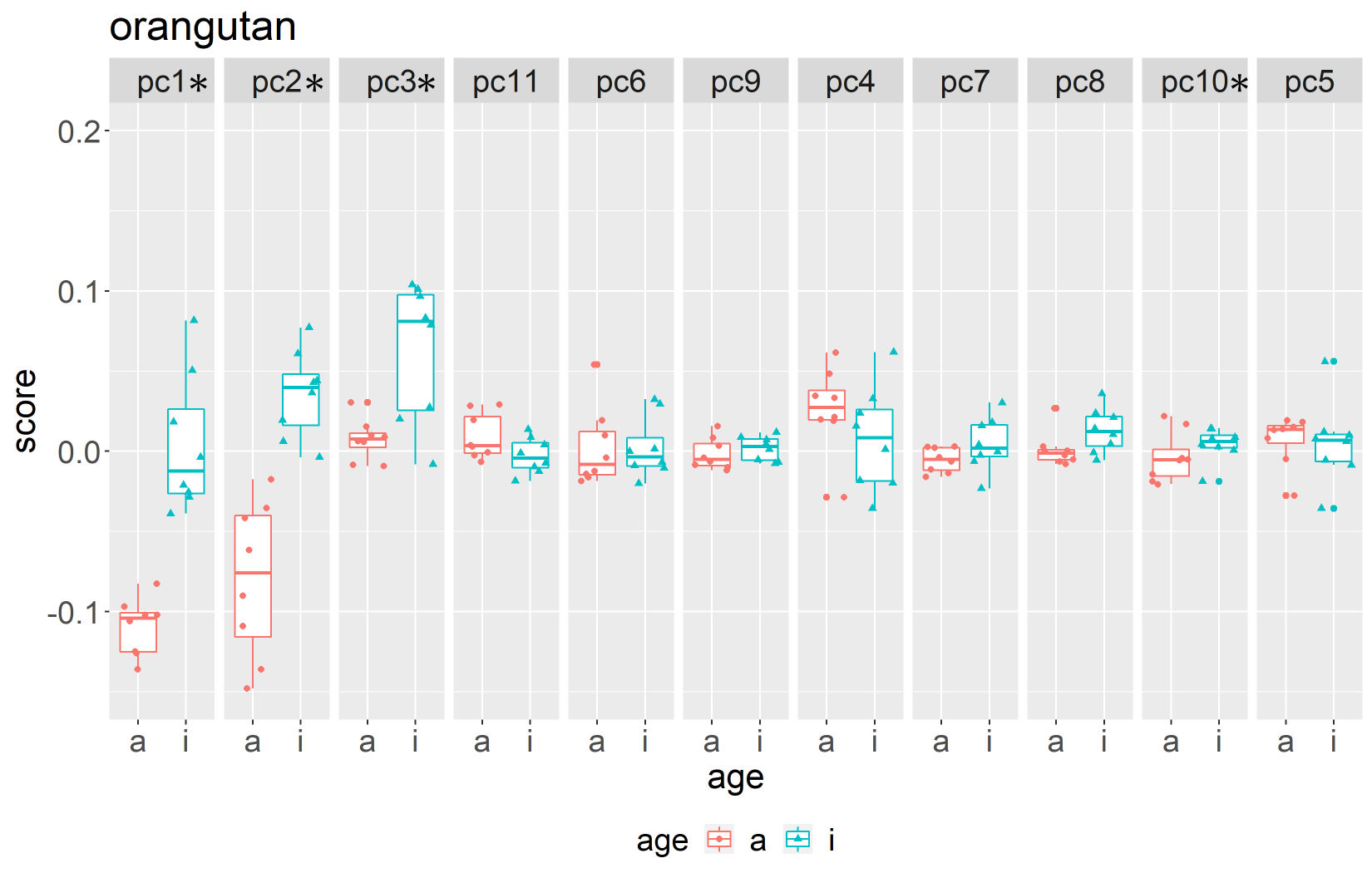


Fig. A.8. PC scores of orangutans.
